# Hypouricemic Effects of Extracts from *Urtica hyperborea* Jacq. ex Wedd. in Hyperuricemia Mice through XOD, URAT1, and OAT1

**DOI:** 10.1155/2020/2968135

**Published:** 2020-01-29

**Authors:** Surina Han, Rongrui Wei, Daorina Han, Jixiao Zhu, Weizao Luo, Wuliji Ao, Guoyue Zhong

**Affiliations:** ^1^Research Center of Natural Resources of Chinese Medicinal Materials and Ethnic Medicine, Jiangxi University of Traditional Chinese Medicine, Nanchang, China; ^2^College of Traditional Mongolian Medicine, Inner Mongolia University for Nationalities, Tongliao, China; ^3^College of Geographical Science, Inner Mongolia Normal University, Hohhot, China; ^4^Chongqing Academy of Chinese Meteria Medica, Chongqing, China

## Abstract

*Urtica* L. has been long used for gout in traditional Tibetan medicine and is closely related to the effect of reducing uric acid. This study aimed to investigate the effect of *Urtica hyperborea* Jacq. ex Wedd. (UW) on lowering uric acid and its mechanism by using HK2 cells and hyperuricemia mouse model. Petroleum ether extract (UWP), ethyl acetate extract (UWE), n-butanol extract (UWB), and alcohol-soluble extract (UWA) from UW were prepared, and HK2 cells were treated with various parts extracts to observe the expression of uric acid transporter at 25, 50, and 100 *μ*g/mL for 24 h. Moreover, hyperuricemia mice were administered orally various parts extracts at 0.78 and 2.34 g/kg (crude drug dose converted by extraction rate) to observe the change of hepatic XOD, serum ADA, renal function, and uric acid transporter. *In vitro* experiments showed that UWA can remarkably elevate OAT1 expression and decrease URAT1 expression in HK2 cells. *In vivo* experiments showed that UWP, UWE, UWB, and UWA showed remarkable activity in reducing uric acid, rendering a substantial decline in the SUA level in hyperuricemia mice. Compared with the hyperuricemia and allopurinol groups, UWB and UWA had significant protective effects on renal injury. At the same time, UWA can significantly reduce the activity of XOD and ADA, reduce the expression of URAT1, and increase the expression of OAT1. These results indicated that UWA had an outstanding uric acid lowering effect and did not affect renal function. This may be related to increased uric acid excretion and decreased uric acid production, mediated by renal OAT1, URAT1, liver XOD, and serum ADA. UWA may be a potential drug against hyperuricemia.

## 1. Introduction

Hyperuricemia is caused by excess uric acid in the blood due to increased production of uric acid and/or impaired renal urate excretion, which is common and extremely painful inflammatory arthritis [1, 2]. It is also an independent risk factor for coronary heart disease, hypertension, diabetes, and other diseases [3]. In recent years, the prevalence of hyperuricemia has been increasing [4]. Currently, there are many drugs used in clinical treatment for lowering uric acid, but there are many side effects, such as allopurinol, mainly headaches, allergies, rashes, elevated aminotransferase, nephritis, and other adverse reactions, contraindication for patients with renal dysfunction. It is necessary to develop effective and low-toxicity drugs against hyperuricemia. Some research has been carried out to find active ingredients of uric acid lowering from traditional Chinese medicine [5, 6].

*Urtica hyperborea* Jacq. ex Wedd. (UW) is a traditional Tibetan medicine, which is a treasure of traditional medicine of China's important ethnic minorities. *U. hyperborea*, a perennial herb, grows in high mountain gravel, rock seam, sand, and grass beach at an altitude of 4000–5200 m and is produced in Tibet, Qinghai, and Sichuan. It is one of the components of the Tibetan medicine “Sazhumu, Shazhu, Shabu” and is recorded in the Tibetan medicine classics “The Four Medical Tantras” and “Jing Zhu Materia Medica.” Its aerial parts, inflorescence, and seed are available for prescription. Importantly, it has been exploited for rheumatoid and rheumatism arthritis for more than 1000 years [7, 8]. Until now, the pharmaceutical research of UW has not been reported, but many studies have shown that other genera of *Urtica* contain a variety of compounds, including flavonoids, alkaloids, lignans, coumarins, terpenoids, steroids, organic acids, volatile oils, and others. Interestingly, most compounds exhibit a variety of biological activities, such as anti-inflammatory, analgesic, antirheumatic, antiprostatic hyperplasia, antibacterial, and antioxidant activities [9]. However, the antigout or antihyperuricemia effects of UW and its potential mechanism have not been reported so far.

In this study, we firstly reported the hypouricemia effects of UW in hyperuricemia mice model established chemically. We prepared petroleum ether extract (UWP), ethyl acetate of extract (UWE), n-butanol extract (UWB), and alcohol-soluble extract (UWA) from UW and tested their activity *in vitro* and *in vivo*. The effects of UWP, UWE, UWB, and UWA on the expression of uric acid transporter protein were detected in HK2 cells. In addition, the effects of UWP, UWE, UWB, and UWA on SUA (serum uric acid) and hepatic XOD (xanthine oxidase) were examined in the hyperuricemia models. BUN (blood urea nitrogen) and creatinine in serum were assayed to evaluate the effects on renal function. In order to further elucidate the underlying mechanisms, the expression levels of the key targets of hyperuricemia, including renal UART1 (uric acid transporter 1) and OAT1 (organic anion transporter 1), were examined.

## 2. Materials and Methods

### 2.1. Materials and Chemicals

Allopurinol, potassium oxonate (PO) and SRB (sulforhodamine B) were obtained from Sigma-Aldrich (St. Louis, MO, USA); uric acid (UA), urea nitrogen (UN), creatinine (Cr), adenosine deaminase (ADA), xanthine oxidase (XOD), and Coomassie brilliant blue protein assay kit were purchased from Nanjing Jiancheng Bioengineering Institute (Nanjing, China). Sodium carboxymethyl cellulose (CMC-Na) was brought from Sinopharm Chemical Reagent Co., Ltd. (Shanghai, China). Fetal bovine serum (FBS) was obtained from Gibco Co., Ltd. (Shanghai, China). RPMI 1640 medium was purchased from Hyclone Co., Ltd. (Shanghai, China). PBS buffer, Penicillin/streptomycin, 0.25% trypsin-EDTA, and enzyme-free water were purchased from Beijing Solarbio Science and Technology Co., Ltd. (Beijing, China). The primary antibodies against rabbit OAT1, URAT1 and the secondary horseradish peroxidase (HRP)-labeled goat-anti-rabbit antibodies were purchased from Abcam Inc (Cambridge, USA). Antibodies against rabbit *β*-actin were acquired from Proteintech Group (Chicago, IL, USA). Other reagents are of analytical grade.

### 2.2. UW Extracts

UW was collected from Pulan and Saga region of Tibet in August 2017. It was identified by Guoyue Zhong researcher of Jiangxi University of Traditional Chinese Medicine. Voucher specimens (XJ077) were preserved in the Research Center of Traditional Chinese Medicine Resources and Ethnic Medicine, Jiangxi University of Traditional Chinese Medicine. UW (9.0 kg) was crushed into powder and extracted with 95% ethanol, 50% ethanol, and water by percolation, respectively. 95% ethanol extract and 50% ethanol extract were extracted until colorless with petroleum ether, ethyl acetate, and n-butanol, successively, merged at the same organic phase, and the extraction solutions were decompressed for recovery. We then obtained petroleum ether extract (UWP, 130 g, extraction yield 1.5%), ethyl acetate of extract (UWE, 30 g, extraction yield 0.33%), and n-butanol extract (UWB, 50 g, extraction yield 0.55%). Water extract was treated with alcohol precipitation and filtered to obtain alcohol-soluble extract (UWA, 900 g, extraction yield 10%).

### 2.3. Cell Culture

Human renal tubular epithelial HK2 cells were purchased from Nanjing Cobioer Biotechnology Co., Ltd. (Nanjing, China). HK2 cells were cultured in RPMI 1640 medium containing 10% FBS, penicillin (100 U/mL), and streptomycin (100 *μ*g/mL) at 37°C with 5% CO_2_. They were routinely subcultured with 0.25% trypsin-EDTA every 3–4 d.

### 2.4. Cell Viability Assay

The viability of HK2 cells was assessed by using the SRB assay. The HK2 cells were exposed to different concentrations (25, 50, and 100 *μ*g/mL) of UWP, UWE, UWB, and UWA for 24 h in 96-well plates, to measure cell viability of the cells. The viability of cultured HK2 cells was assessed by SRB method (Osman et al. 2012). Briefly, HK2 cells were seeded in 96-well plates at a cell density of 5 × 10^3^ cells/well. After 24 h incubation, the cells were treated with varying concentrations of UWP, UWE, UWB, and UWA (25, 50, and 100 *μ*g/mL), respectively. Drugs were added either in a simultaneous for 24 h. Cells were fixed in situ by adding 100 *μ*L of cold 10% TCA for 1 h at 4°C. Then discard the supernatant, rinse plate with distilled water, dry it, dye it with SRB dissolved in 1% acetic acid for 30 min at room temperature, rinse it with 1% acetic acid and dry it, and add 10 mM Tris base (PH 10.5) with 100 *μ*L/well to dissolve the dye for 10 min; the optical density (OD) was measured by spectrophotometer (Thermo Fisher Scientific Oy, Vantaa, Finland) at 515 nm.

### 2.5. Western Blot Analysis

Cell lysates were prepared, and protein concentration was quantified using the BCA assay kit (Beijing Dingguo Changsheng Biotechnology CO., Ltd. Beijing, China). Equal amounts of proteins were separated with 10% sodium dodecyl sulfate (SDS)-polyacrylamide gels and then transferred onto PVDF membranes (Millipore CO., Ltd. Beijing, China). The membranes were blocked in Tris-buffered saline/Tween 20 (TBS-T) containing 5% skim milk for 1 h and incubated with primary antibodies against URAT1, OAT1, and *β*-actin overnight at 4°C. After washing with the TBS-T buffer, the membrane was incubated with the secondary antibody. Specific bands were visualized on gel imager (Bio-rad, USA) by chemiluminescence using ECL™ Detection reagents (Abbkine Biotechnology Co., Ltd., Wuhan, China).

### 2.6. Hyperuricemia Mice and Drug Administration

Male KM mice (18 ± 2 g) were obtained from Hunan SJA Laboratory Animal Co., Ltd. (SCXK2016-0002) (Hunan, China). They were allowed to adapt to the experimental environment for one week. PO was used as a powerful inhibitor of uricase to induce the hyperuricemia mice model. Mice (110) were randomly divided into eleven groups: control, hyperuricemia (PO, 350 mg/kg), allopurinol (10 mg/kg), and eight drug groups with UWP, UWE, UWB, and UWA at low and high dose (UWP-L, UWP-H, UWE-L, UWE-H, UWB-L, UWB-H, UWA-L, and UWA-H), wherein PO (350 mg/kg) was injected for elevating SUA intraperitoneally. Then, control and hyperuricemia groups were given saline. The allopurinol group was treated with allopurinol at 10 mg/kg. UWP, UWE, UWB, and UWA were administered in the low and high dose groups (0.78 and 2.34 g/kg, crude drug dose converted by extraction yield), respectively. All groups were given continuous gavage for 7 days, and hyperuricemia mice model was established by intraperitoneal injection of PO 1 h before the final gavage. One hour after the last gavage, the mice were killed and serum, liver, and kidney were collected to perform biochemical analysis and RT-PCR analysis. All experimental procedures were approved by the Guidance Suggestions for the Care and Use of Experimental Animals of the Jiangxi University of Traditional Chinese Medicine. Ethic number of animal the study is JZLLSC2018-0093.

### 2.7. Biochemical Analysis of Hyperuricemic Mice

The serum sample was assayed to determine the levels of UA, creatinine (Cre), blood urea nitrogen (BUN), and adenosine deaminase (ADA) according to the manufacturer's instructions, respectively. Liver XOD activities were examined using the XOD assay kit in accordance with the manufacturer's protocol.

### 2.8. RT-PCR Analysis of URAT1 and OAT1 in the Kidney of Hyperuricemia

The PCR amplification primers were designed as described in reference Fang et al. [10], as shown in Table 1. The kidney cortex tissue weighed 50–100 mg, and the total RNA was extracted using TRIzol reagent (Ambion CO., Ltd., USA). After homogenization of the kidney cortex tissue, the obtained liquid was mixed with 200 *μ*L chloroform and centrifuged, followed by precipitating the aqueous phase with an equivalent volume of isopropanol. After washing with 1 mL ethanol (75%), the total RNA pellets were suspended using DEPC water. The reverse transcription was conducted using 1 *μ*g RNA with reverse transcriptase (Shanghai Novoprotein Technology Co. Ltd., Shanghai, China). The obtained cDNA was diluted with DNase-free water and PCR amplification was performed in 30 cycles of 94°C for 90 s, then 94°C for 20 s, 57°C for 20 s, and 72°C for 60 s. The final elongation step was 72°C for 5 min, and was kept warm at 4°C. *β*-actin was used as an endogenous standard. Finally, the PCR products were quantified by electrophoresis.

### 2.9. Statistical Analysis

All the presented data are expressed as mean ± SD. Statistical analysis was performed using a one-way analysis of variance (ANOVA) by using SPSS 19.0 software (SPSS, Inc., Chicago, IL, USA). The difference was considered statistically significant when *P* < 0.05.

## 3. Results

### 3.1. Effects of Different Extracts from UW on Uric Acid Transporters in HK2 Cells

**Figure 1:**
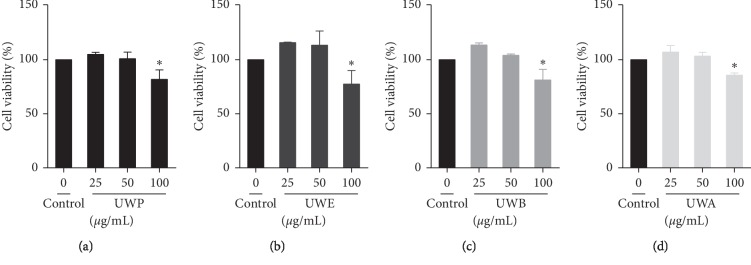
Effect of different extracts from UW on cell viability of HK2 cells. The cells were treated with the indicated concentrations of UWP (a), UWE (b), UWB (c), and UWA (d) for 24 h. Cell viability was determined by the SRB assay. Values are expressed as mean ± SD from three independent replicates. ^*∗*^*P* < 0.05 compared with the control group.

**Figure 2:**
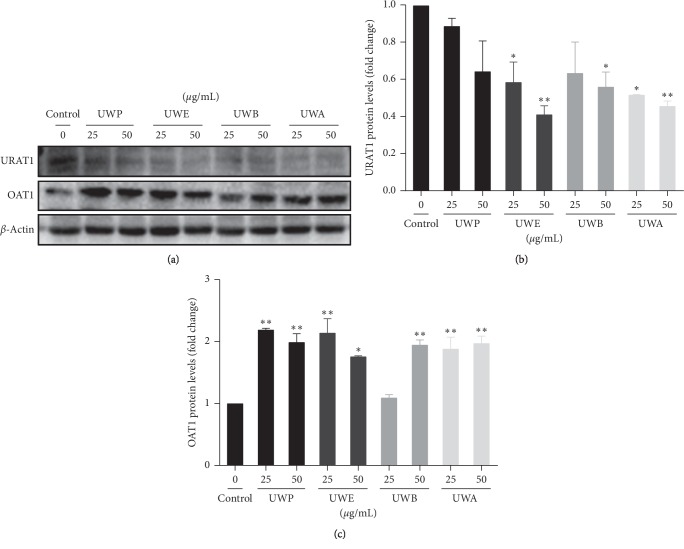
Effects of different extracts from UW on URAT1 and OAT1 expression in HK2 cell. The cells were treated with the indicated concentrations of UWP, UWE, UWB, and UWA for 24 h, respectively. The protein expression levels of URAT1 and OAT1 were analyzed via western blotting. *β*-Actin was used as a loading control, and blots are representative of at least three independent experiments. Each bar represents the mean ± SD (*n* = 3).^*∗*^*P* < 0.05, ^*∗∗*^*P* < 0.01 compared with the control group.

In this study, the effects of different extracts from UW on cell viability were measured by SRB assay to determine the optimal concentration of different extracts from UW (UWP, UWE, UWB, and UWA). Cell viability was not affected at 25 and 50 *μ*g/mL of UWP (Figure 1(a)), UWE (Figure 1(b)), UWB (Figure 1(c)), and UWA (Figure 1(d)), respectively. At 100 *μ*g/mL, different extracts from UW significantly reduced cell survival rate. Therefore, 25 *μ*g/mL and 50 *μ*g/mL were used to study the effects of different extracts from UW on the uric acid transporter in HK2 cells. Effects of different UW extracts on URAT1 and OAT1 expression in HK2 cells. When the HK2 cells were incubated with UWP, UWE, UWB, and UWA at 25 and 50 *μ*g/mL for 24 h, compared with the control group, UWE (25 *μ*g/mL), UWB (50 *μ*g/mL), and UWA (25 *μ*g/mL) decreased the expression of URAT1 protein (*P* < 0.05) and UWE (50 *μ*g/mL) and UWA (50 *μ*g/mL) significantly downregulated URAT1 protein expression (*P* < 0.01) (Figures 2(a) and 2(b)). Moreover, UW extracts can upregulate the expression of OAT1 protein, especially, UWP (25 and 50 *μ*g/mL), UWE (25 *μ*g/mL), UWB (50 *μ*g/mL), and UWA (25 and 50 *μ*g/mL) which significantly upregulated the expression of OAT1 protein (*P* < 0.01) (Figures 2(a) and 2(c)).

### 3.2. Different Extracts from UW Reduced SUA Levels in Hyperuricemia Mice

**Figure 3:**
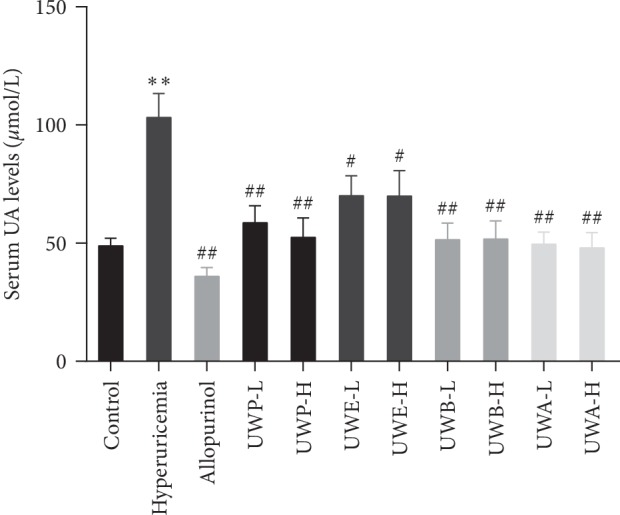
Effects of UW extracts on the level of SUA in hyperuricemia mice. Uric acid was examined based on the phosphotungstic acid reaction. Data was expressed as mean ± SD; *n* = 10. ^*∗∗*^*P* < 0.01, compared to the control group; ^#^*P* < 0.05, ^##^*P* < 0.01, compared to the hyperuricemia group.

The hypouricemia activities of different extracts from UW were assessed by assaying the level of SUA in hyperuricemia mice. The models were established successfully by injecting oxonic acid. As shown in Figure 3, compared with the control group, the serum UA level in hyperuricemia group significantly increased after PO administration (*P* < 0.01). Allopurinol, as a positive control drug, significantly decreased UA level in serum compared with hyperuricemia group mice (*P* < 0.01). Compared with the hyperuricemia group, different doses of UW extracts can reduce the UA level. In particular, UWP, UWB, and UWA significantly reduced UA content at high and low doses (*P* < 0.01). Similar to allopurinol, the UWP, UWB, and UWA groups were close to the control group. Therefore, UWP, UWB, and UWA have good effect on lowering uric acid in hyperuricemia mice.

### 3.3. Different Extracts from UW Improved Renal Function

**Figure 4:**
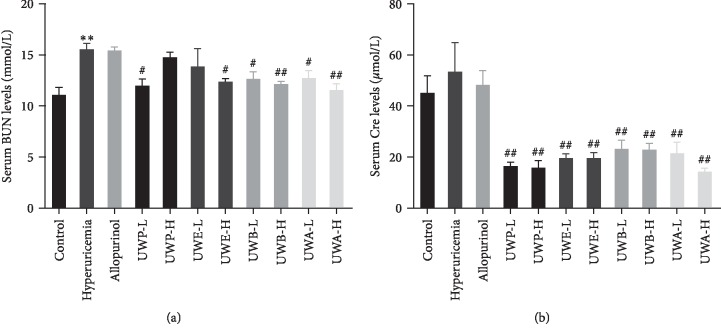
Effects of UW extracts on the level of BUN (a) and SCr (b) in hyperuricemia mice. Data was expressed as mean ± SD, *n* = 10. ^*∗∗*^*P* < 0.01, compared to the control group; ^#^*P* < 0.05, ^##^*P* < 0.01, compared to the hyperuricemia group.

BUN and Cre were used to evaluate renal function. The hyperuricemia group exhibited BUN at 15.54 mmol/L, which was higher than that of the control group (11.08 mmol/L, *P* < 0.01, Figure 4(a)). BUN in the allopurinol group (15.41 mmol/L) was similar to that in the hyperuricemia group. Groups with UW extracts decreased the BUN levels, especially, UWB-H and UWA-H which can reduce BUN levels to 12.12 and 11.54 mmol/L (*P* < 0.01), respectively, similar to the control group. Moreover, compared to the control group, the level of Cre in the hyperuricemia group was higher than that in the normal group, but the change of Cre was not statistically significant (Figure 4(b)), indicating mild renal function damage. Compared to the hyperuricemia group, 0.78 and 2.34 g/kg of UWP, UWE, UWB, and UWA reduced Cre levels, while allopurinol treatment failed to reduce Cre (Figure 4(b)).

### 3.4. UW Extracts Decreased the Activity of Liver XOD and Serum ADA

**Figure 5:**
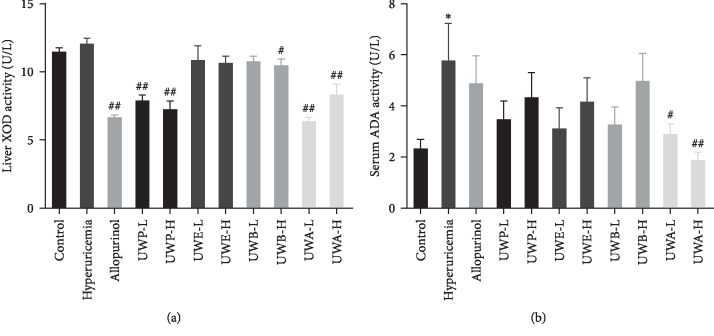
Effects of UW extracts on the activity of hepatic XOD (a) and serum ADA (b) in hyperuricemia mice. Data was expressed as mean ± SD; *n* = 10. ^*∗*^*P* < 0.05, compared to the control group; ^#^*P* < 0.05, ^##^*P* < 0.01, compared to the hyperuricemia group.

XOD and ADA are important enzymes in uric acid production. To investigate the effects of UW extracts on XOD and ADA, the activity of liver XOD and serum ADA in mice was checked. Compared to the control group, hepatic XOD activity of the model group was higher, but there was no statistical significance (Figure 5(a)). Compared to the hyperuricemia group, liver XOD activity of the UWP-L, UWP-H, UWB-H, UWA-L, UWA-H, and allopurinol groups was significantly reduced. Simultaneously, serum ADA was dramatically increased in the hyperuricemia group (Figure 5(b)). UWA at 0.78 and 2.34 g/kg notably lowered the ADA activities in serum. The effect of UWA is better than that of allopurinol in the aspect of reducing ADA activity. These data indicated that UW extracts inhibited XOD and ADA activity to reduce uric acid production, and UWA exhibited potent inhibition of XOD and ADA *in vivo*.

### 3.5. Effects of UW Extracts on Uric Acid Transporters URAT1 and OAT1 in Kidneys of Hyperuricemia Mice

**Figure 6:**
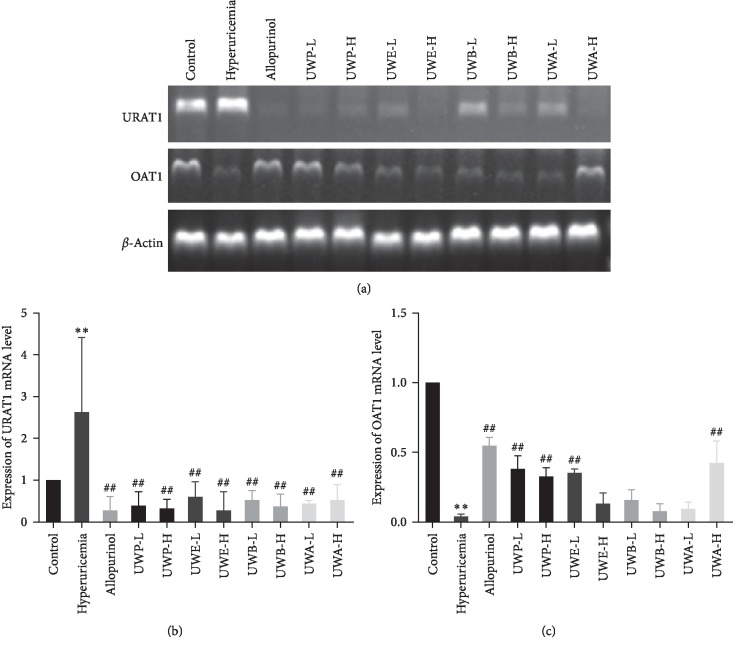
Effects of UW extracts on mRNA levels of renal urate transporters OAT1 and URAT1 (a) in mice. Data were normalized to *β*-actin and expressed as relative fold (b, c). ^*∗∗*^*P* < 0.01, compared to the control group; ^##^*P* < 0.01, compared to the hyperuricemia group.

Renal URAT1 mRNA level was remarkably increased in the hyperuricemia group, and OAT1 mRNA level was significantly decreased in the hyperuricemia group (Figure 6(a)). Compared with the hyperuricemia group, UWP, UWE, UWB, and UWA groups downregulated the mRNA levels of URAT1 at low and high doses, respectively (Figure 6(b)), and the groups of UWP-L, UWP-H, UWE-L, and UWA-H had significantly increased OAT1 mRNA levels (Figure 6(b)). These results indicated that UWP-L, UWP-H, UWE-L, and UWA-H groups downregulated the expression of URAT1 in renal tissues, upped OAT1 expression, and promoted the excretion of uric acid.

## 4. Discussion

There are abundant resources of nettle plants in China, which have a long history in traditional medicine and folk medicine [11]. *Urtica* has long-term clinical application foundation and broad development prospects in the treatment of diabetes, hypertension, rheumatoid arthritis, prostatic hyperplasia, and other major diseases [12]. Presently, we described the UW, one plant of the genus nettles, used for ameliorating hyperuricemia. Hyperuricemia and its related diseases are prevalent, especially gout [13], risking human life globally. Therefore, after cancer, diabetes, and hyperlipidemia, hyperuricemia has received great focus. However, the choice of treatment for hyperuricemia is limited because some commonly prescribed drugs such as allopurinol and benzbromarone have a series of side effects [14, 15]. Current research is also focused on this point. This study proved for the first time that UW extracts have the effects of reducing uric acid and protecting the kidney in mice with hyperuricemia.

Uric acid (UA) elevation in the human body is mainly caused by purine metabolism disorder and/or uric acid excretion disorder. 70% of uric acid in the body is excreted by the kidneys with urine [16]. Hyperuricemia is the biochemical basis for gout, which must be accompanied by hyperuricemia [8]. Studies have shown that in mainland China, the pooled prevalence of hyperuricemia and gout is 13.3% and 1.1%, respectively [17]. In this study, SUA level in the hyperuricemia model group was significantly increased compared with the control group, indicating that the modeling was successful. In addition, it is believed that PO could seriously impair renal function. In this experiment, the BUN and Cre levels increased in the serum of hyperuricemia group. These are in line with previous reports [6]. After a week of treatment with different extracts from UW, compared with hyperuricemia group, allopurinol and UW extract groups had the effect of decreasing SUA level, in particular, high and low doses of UWA extract can not only reduce uric acid level but also can reduce the level of Cre and BUN.

Many studies showed that the rate-limiting enzymes that catalyze UA production included XOD, ADA, and so on. XOD is an existing form of xanthine oxidase (XOR) in the body and is mainly synthesized in the liver [18]. It is mainly synthesized in the liver and is a key enzyme for uric acid production and catalyzes hypoxanthine and xanthine to eventually produce uric acid. Adenosine is catalyzed by ADA to produce inosine, which ultimately produces hypoxanthine [18]. Therefore, it is also one of the important indicators in the study of antihyperuricemia. This study shows that the UWP and UWA groups with high and low doses had significant inhibitory effects on liver XOD activity, similar to the allopurinol group. Only low and high doses of UWA can remarkably inhibit ADA activity. These results are inconsistent with the effect of the same dose group on SUA level, indicating that there are other mechanisms to reduce uric acid, such as the role of uric acid transporters.

Uric acid transporters OAT1 and URAT1 were identified as potential therapeutic targets for hyperuricemia [6]. Previous studies have shown that 70% of urate is mainly dependent on the renal urate transport system [19]. Our *in vitro* experiments on HK2 cells show that different extracts from UW had different regulatory effects on OAT1 and URAT1. These results suggest that UW extracts may modulate renal uric acid transporter in hyperuricemia mice. As expected, UWP, UWE, UWB, and UWA at high and low dose groups significantly reduced the expression of URAT1 in hyperuricemia mice, while UWP-L, UWP-H, UWE-L, and UWA-H remarkably increased the expression of OAT1. In this study, both *in vitro* and *in vivo* experiments showed that UW extracts had different degrees of regulatory effects on uric acid transporters, and most of the results were consistent. Therefore, the results of *in vitro* experiments can provide certain reference and guidance for *in vivo* experiments. It suggested that uric acid transporter inhibitors could be screened by HK2 cells *in vitro*. Different extracts of UW can regulate the expression of uric acid transporter, which may be one of its mechanisms to reduce uric acid.

From the results of the study, UWA exhibits the dual effect in inhibiting XOD and regulating uric acid transporters, but it is unclear which mechanism is more important. We speculate that it contains both XOD inhibitors and URAT1 inhibitors. At present, we only separated the monomer compounds can reduce the uric acid transporter in vitro cell experiments, but no XOD inhibitory activity (the data have not yet been published). However, the experimental results showed that the extract had the activity of affecting both uric acid transporter and XOD, and it was speculated that the extract contained two components of the action mechanism, but a lot of further work was needed to confirm this. In addition, compared with the group allopurinol, the UWA also reduced the activity of ADA and had protective effect on renal function.

## 5. Conclusions

We firstly evaluate the effect and mechanism of UW extracts in reducing uric acid in in vivo and in vitro experiments. The results showed that all extracts from UW decreased SUA level and regulated the expression of uric acid transporter URAT1. Some extracts decreased the activity of XOD and ADA and increased the expression of OAT1. In addition, UWB-H and UWA-H exhibit a significantly protective effect on the renal injury of PO-induced hyperuricemia mice, in which UWA can simultaneously reduce the activity of XOD and ADA and regulate the expression of uric acid transporter. Therefore, the mechanism of UWA reducing uric acid may be to regulate the activities of XOD and ADA, and the expression of uric acid transporter (URAT1 and OAT1). UWA, as an active fraction of UW, may contain active compounds against hyperuricemia, which need to be further studied. The current results provided an important screening method for antiuric acid active compounds and a theoretical basis for the uric acid lowering effect of UW.

## Figures and Tables

**Table 1:** PCR primer sequences.

Gene	Sequence (5′-3′)	Product size (bp)
URAT1	Forward: GCTACCAGAATCGGCACGCT	343
	Reverse: CACCGGGAAGTCCACAATCC	
OAT1	Forward: ACGGGAAACAAGAAGAGGG	580
	Reverse: AAGAGAGGTATGGAGGGGTAG	
*β*-Actin	Forward: GTCACCAGGGTGTCATGGTA	105
	Reverse: GTCCCAGTTGGTGATGATGC	

## Data Availability

Table 1 and Figures 1–6 data used to support the findings of this study are included within the article.

## References

[1] Mancia G., Grassi G., Borghi C. (2015). Hyperuricemia, urate deposition and the association with hypertension. *Current Medical Research and Opinion*.

[2] Zhu Y., Pandya B. J., Choi H. K. (2012). Comorbidities of gout and hyperuricemia in the US general population: NHANES 2007-2008. *The American Journal of Medicine*.

[3] Sato V. H., Sungthong B., Rinthong P.-O. (2018). Pharmacological effects of chatuphalatika in hyperuricemia of gout. *Pharmaceutical Biology*.

[4] Hou C.-W., Lee Y.-C., Hung H.-F., Fu H.-W., Jeng K.-C. (2012). Longan seed extract reduces hyperuricemia via modulating urate transporters and suppressing xanthine oxidase activity. *The American Journal of Chinese Medicine*.

[5] Yong T., Zhang M., Chen D. (2016). Actions of water extract from *Cordyceps militaris* in hyperuricemic mice induced by potassium oxonate combined with hypoxanthine. *Journal of Ethnopharmacology*.

[6] Yong T., Chen S., Xie Y. (2018). Hypouricemic effects of *Armillaria mellea* on hyperuricemic mice regulated through OAT1 and CNT2. *The American Journal of Chinese Medicine*.

[7] Chinese Flora Editorial Board of the Chinese Academy of Sciences (1993). *Flora of China*.

[8] Feng X., Wang M., Cheng J., Li X. (2017). Two new secolignans with in vitro anti-inflammatory activities from Urtica fissa rhizomes. *Journal of Natural Medicines*.

[9] Su R., Luo W., Zhu J. (2018). Research progress on medicinal plant of *Urtica L*. *Chinese Traditional and Herbal Drugs*.

[10] Fang C., Chen L., He M. (2019). Molecular mechanistic insight into the anti-hyperuricemic effect of Eucommia ulmoides in mice and rats. *Pharmaceutical Biology*.

[11] Chen X., He S., Lu Y. (2015). Inhibition of spontaneous canine benign prostatic hyperplasia by an *Urtica fissa* polysaccharide fraction. *Planta Medica*.

[12] Feng B., Yan X., Wang H., Shi L., Tang L., Wang Y. (2010). Two new secolignan glycosides from the roots of Urtica triangularis Hand.-Mazz. *Fitoterapia*.

[13] Yang H., Gao L., Niu Y. (2015). Mangiferin inhibits renal urate reabsorption by modulating urate transporters in experimental hyperuricemia. *Biological & Pharmaceutical Bulletin*.

[14] Maureen D., Zhu Y., Zhang Y. (2014). Allopurinol initiation and all-cause mortality in the general population. *Annals of the Rheumatic Diseases*.

[15] Azevedo V. F., Buiar P. G., Giovanella L. H., Severo C. R., Carvalho M. (2014). Allopurinol, benzbromarone, or a combination in treating patients with gout: analysis of a series of outpatients. *International Journal of Rheumatology*.

[16] Kou Y., Li Y., Ma H. (2016). Uric acid lowering effect of Tibetan Medicine RuPeng 15 powder in animal models of hyperuricemia. *Journal of Traditional Chinese Medicine*.

[17] Rui L., Cheng H., Di W. (2015). Prevalence of hyperuricemia and gout in mainland China from 2000 to 2014: a systematic review and Meta-Analysis. *BioMed Research International*.

[18] Pang M., Fang Y., Chen S. (2017). Gypenosides inhibits xanthine oxidoreductase and ameliorates urate excretion in hyperuricemic rats induced by high cholesterol and high fat food (lipid emulsion). *Medical Science Monitor*.

[19] Pascual E., Perdiguero M. (2006). Gout, diuretics and the kidney. *Annals of the Rheumatic Diseases*.

